# Cardiovascular risk factors and autonomic indices in relation to fatal and non-fatal coronary events

**DOI:** 10.1136/openhrt-2020-001445

**Published:** 2021-04-20

**Authors:** Christian Zambach, Artur Fedorowski, Yan Borné, Linda S B Johnson, Sofia Gerward, Viktor Hamrefors, Gunnar Engström

**Affiliations:** 1 Department of Clinical Sciences, Lund University, Malmö, Sweden; 2 Department of Internal Medicine, Skåne University Hospital, Lund, Sweden; 3 Department of Cardiology, Skåne University Hospital, Malmö, Sweden; 4 Department of Medical Imaging and Physiology, Skåne University Hospital, Malmö, Sweden; 5 Department of Internal Medicine, Skåne University Hospital, Malmö, Sweden

**Keywords:** coronary artery disease, acute coronary syndrome, risk factors

## Abstract

**Objective:**

Mortality caused by coronary artery disease has markedly decreased in recent years. However, a substantial proportion of patients suffering a coronary event (CE) die within the first day, most of them out of hospital. We aimed to investigate how established cardiovascular (CV) risk factors and CV autonomic indices associate with fatal versus non-fatal CEs in the population.

**Methods:**

33 057 individuals (mean age; 45.6 years; 10 773 women) free of coronary artery disease at baseline were included. Baseline examination, including assessment of traditional CV risk factors and autonomic indices such as heart rate and orthostatic reaction, was performed during 1974–1992, after which the subjects were monitored for incident CV disease. The Lunn-McNeil competing risks approach with a prespecified multivariable model was used to assess differences in risks for fatal and non-fatal CEs in relation to baseline CV risk factors.

**Results:**

During follow-up period of 29.7 years, 5494 subjects (6.10/1000 person-years) had first CE; 1554 of these were fatal. Age, male gender, smoking, body mass index (BMI), blood pressure, pulse pressure and resting heart rate had stronger relationships with fatal CE than with non-fatal events. The effects of diabetes, serum cholesterol, antihypertensive treatment and orthostatic blood pressure responses were similar for fatal and non-fatal CE.

**Conclusions:**

Several cardiovascular risk factors, such as smoking, high BMI, blood pressure and high resting heart rate, were preferentially associated with fatal compared with non-fatal CEs. These observations may require special attention in the overall efforts to further reduce coronary artery disease mortality.

**Key questions**

**What is already known about this subject?**

- In recent decades, intensive research has led to the identification of several well-established risk factors for coronary artery disease, including acute coronary events (CEs) such as myocardial infection. It is well known that a substantial proportion of subjects who suffer an acute CE die outside the hospital and that sudden cardiac death can be the first sign of coronary artery disease. Identification of subjects at risk of a fatal first-time CE remains a major challenge in cardiovascular prevention.

**What does this study add?**

- We have linked risk factor data from a public health initiative conducted in 1974–1992 with incident fatal and non-fatal CEs, retrieved from several registers, during follow-up until the 31 December 2013. We report that certain established cardiovascular risk factors as well as increased resting heart rate, as indices of cardiovascular autonomic dysfunction, are preferentially associated with higher risk of fatal outcome in a first-time CE.

**How might this impact on clinical practice?**

- These risk factors, including indices of cardiovascular autonomic dysfunction, may require special attention in cardiovascular prevention programmes and in the overall efforts to further reduce coronary artery disease mortality.

## Introduction

In recent decades, better control of cardiovascular risk factors and improved acute treatments have markedly improved the prognosis for patients with acute coronary events (CEs), including myocardial infarction.^1^ However, coronary artery disease still remains the major cause of mortality globally.^2^ It is well known that a substantial proportion of subjects who suffer an acute CE die outside the hospital and that sudden cardiac death can be the first sign of coronary atherosclerosis.^3–5^ Identification of subjects at risk of a fatal first-time CE remains a major challenge in cardiovascular prevention.^6^

Blood pressure, smoking, diabetes, blood lipids and obesity are all well-established risk factors for acute CEs.^7 8^ Only few studies, however, have explored whether these risk factors are related to the fatality of first-time CE.^1 9 10^ Male gender, age, hypertension, diabetes and inflammation have been reported to be more strongly associated with fatal CE, as compared with non-fatal events.^9–13^ In contrast, hyperlipidaemia has been reported to be similarly associated with fatal and non-fatal CE,^9 10^ whereas data for smoking in relation to fatal CE has been inconsistent.^9 10^

Beside established cardiovascular disease (CVD) risk factors, increasing evidence suggests that cardiovascular autonomic dysfunction, manifested for example by increased resting heart rate, reduced heart rate variability or orthostatic hypotension, is a risk factor for CEs.^14–16^ Previous studies have reported relationships between orthostatic hypotension and several adverse cardiovascular outcomes,^14 16^ but it is not known whether CV autonomic indices and orthostatic hypertension in particular are related to CE fatality.

The purpose of the present study was to determine which established cardiovascular risk factors are preferentially associated with fatal as compared with non-fatal first-time CE in the population. In addition, we tested if factors related to autonomic function, including increased resting heart rate and an impaired orthostatic blood pressure response, were more strongly associated with fatal first time CE.

## Methods

### Study population

A voluntary screening programme with the aim to detect individuals at high risk for CVD, the Malmö Preventive Project, was conducted in Malmö, Sweden, between 1974 and 1992. The screening programme has previously been described in detail.^17^ In summary, complete births cohorts, born between 1921 and 1949, were invited to a health examination, including physical examination, a panel of laboratory blood tests and a self-administered questionnaire. A total of 22 444 men and 10 902 women attended and were examined from 1974 to 1992, yielding an overall participation rate of 71%. Men were mostly screened in the first half of the period (1974–82), and women in the latter half (1981–1992), explaining the different numbers and the different length of follow‐up time. Subjects with unfavourable risk factors at baseline were offered interventions, as has been previously described.^17^ The study complied with the Declaration of Helsinki. Informed consent was obtained from all subjects.

For the current study, we excluded subjects with a history of a CE (n=122) and those with missing information about systolic blood pressure (n=45), body mass index (BMI) (n=14), cholesterol (n=80), antihypertensive treatment (n=51) and smoking habits (n=53). The final cohort thus consisted of 33 057 individuals (22 284 men and 10 773 women). There was some additional random missing data for orthostatic blood pressure tests (n=481) and resting heart rate measurements (n=133), and the separate analyses of these variables are therefore based on slightly smaller samples.

### Baseline examinations

Blood pressure (mm Hg) was measured in the right arm using a sphygmomanometer and a rubber cuff of appropriate size placed at the heart level after 10 min of supine rest, and the average of two measurements was recorded. Height (cm) and weight (kg) were measured under standardised conditions in light indoor clothing. BMI was calculated as weight/height^2^ (kg/m^2^). The subjects were categorised as smokers or non-smokers based on self-reported current smoking. Blood samples were drawn after overnight fast. Serum cholesterol and fasting blood glucose were analysed using standard methods at the hospital laboratory.

Diabetes was defined as either fasting whole blood glucose ≥6.1 mmol/L (corresponding to plasma glucose ≥7.0 mmol/L), self-reported diabetes or a prevalent diagnosis of diabetes in the Swedish hospital discharge register.

Orthostatic blood pressure reactions were measured as follows: the first blood pressure denotes an average of two measurements after 10 min supine rest. The second blood pressure denotes an average of two measurements after 1 min of standing. All measurements were rounded to nearest 5 mm Hg. Orthostatic blood pressure decrease was defined as the average standing blood pressure minus the average values in the supine position (ie, a positive value corresponds to a blood pressure decrease on standing). Resting heart rate was measured twice by palpation of the radial artery over 1 min in the supine position. The mean of two measurements were recorded as beats per minute (bpm).

Orthostatic hypotension was defined according to the international consensus as a decrease in systolic blood pressure of 20 mm Hg or more or a decrease in diastolic blood pressure of 10 mm Hg or more.^18^ Antihypertensive treatment was defined as a positive answer to the question ‘Do you take medication for high blood pressure?’.

### Patient and public involvement

The included subjects for the current study were all part of the Malmö Preventive Project, which was a population screening programme in Malmö, Sweden, with the aim to detect individuals at high risk for CVD. The subjects were included between 1974 and 1992. At the time, the subjects went through a health examination, including physical examination, a panel of laboratory blood tests and a self-administered questionnaire. Subjects with unfavourable risk factors detected during the screening programme were offered preventive interventions as has been previously described.^17^

### Endpoint retrieval

Follow-up was from baseline until a first incident CE, or censoring due to death, emigration or 31 December 2013. Besides emigration (1.9 %), there was no loss to follow-up. The endpoint was defined as a CE (International Classifications of Diseases (ICD) codes 410–414 in ICD-9 and codes I20-I25 in ICD-10) retrieved from the Swedish National Hospital Discharge Register (current Swedish Patient Register) or the Cause of Death Register. A fatal CE was defined as death on the same day as the first diagnosis of CE, inside or outside of a hospital.^12 19^ Of the 1554 fatal CEs, cause of death was based on autopsy in 1015 (65.3%) cases. The findings from the current study were based on data linked after the study outcomes.

### Statistical analysis

In order to examine the preferential association between different risk factors at baseline and fatal first time CE, we used the Lunn-McNeil adaptation of Cox proportional hazards regression. This method has been used for similar purposes elsewhere.^20 21^ Briefly, the method consists of duplicating the dataset so that each individual occurs in two strata. The separate endpoints, fatal and non-fatal CE, are separated into these strata. All covariates are also duplicated but set to the value 0 in one of the strata according to the endpoint. This allows for the modelling of the separate failure-specific effect of each variable in a stratified Cox regression, with stratification on failure strata. The effect measures derived from these stratified Cox regressions are identical to what would have been obtained in a standard Cox regression for each failure. The p value for equal effects across failure types is then derived using the likelihood ratio test, after running a model where only one variable is unduplicated and thus forced to have the same effect measure for both failure types. The proportional hazards assumption was tested visually by plotting the CE-free survival over time in the unduplicated dataset for each failure type.

We used two prespecified models. Model 1 adjusted for age and gender. Model 2 included seven additional established cardiovascular risk factors: age, gender, diabetes, current smoking, cholesterol levels, BMI, antihypertensive treatment and systolic or diastolic blood pressure per 10 mm Hg.

We tested for interactions between gender and other risk factors by adding interaction terms to the Cox regression analyses, with adjustments in model 2. The interaction term was statistically significant for gender and smoking for non-fatal events, indicating a stronger association between smoking and non-fatal CEs in women than in men. However, the effect of this interaction on the effect measures for the other risk factors was small, and the interaction parameter was left out of the final model. A p value less than 0.05 was considered significant. Analyses were performed using IBM SPSS V.25 64-bit edition (IBM) and STATA V.12.0 SE (StataCorp).

## Results

### Baseline characteristics

About two-thirds of the study cohort were men and 44.7% were smokers. Women were more often treated with antihypertensive drugs, had a slightly higher prevalence of diabetes at baseline and were less often current smokers (35% compared with 51% in men). The prevalence of orthostatic hypotension at baseline was 7.9% in women and 5.2% in men (table 1).

**Table 1:** Baseline characteristics

Baseline characteristics of the study population	All	Female	Male
Number of subjects	33 057	10 773	22 284
Age, years	45.6 (7.4)	49.6 (7.4)	43.7 (6.6)
Gender, % male	67.3	0	100
BMI, kg/m^2^	24.6 (3.6)	24.4 (4.2)	24.7 (3.3)
Current smoker, %	44.7	35.3	50.8
Supine SBP, mm Hg	126.3 (15.5)	124.7 (16.7)	127 (14.9)
Supine DBP, mm Hg	84.2 (9.6)	81.6 (9.1)	85.5 (9.6)
Pulse pressure, mm Hg	42 (10.9)	43 (11.6)	41.6 (10.5)
Postural SBP response, mm Hg	−1.2 (8.7)	−2.6 (9)	−0.5 (8.5)
Postural DBP response, mm Hg	2.2 (5.3)	1.6 (5.2)	2.5 (5.4)
Orthostatic hypotension, %	6.1	7.9	5.2
Resting heart rate, bpm	67.5 (9.7)	68 (9)	67 (10)
Diabetes, %	3.5	3.8	3.3
Cholesterol, mmol/L	5.7 (1.1)	5.8 (1.1)	5.6 (1.19
Antihypertensive treatment, %	5.4	8.4	3.9
Incidence of non-fatal coronary events, per 1000 person years	4.4	2.76	5.11
Incidence of fatal coronary events, per 1000 person years	1.7	0.86	2.12

Values displayed as mean (SD) if not otherwise indicated.

BMI, body mass index; bpm, beats per minute; DBP, diastolic blood pressure; SBP, systolic blood pressure.

### Incidence of fatal and non-fatal CEs

The median follow-up time from baseline to a first CE, death, emigration or last follow-up date was 29.7 years (IQR 10.9). During the follow-up period, there were 5494 first-time CEs (6.10 per 1000 person-years). Of these, 1554 (28.3%) were fatal. In men, the incidence of fatal and non-fatal CEs was 2.12 and 5.11 per 1000 person years, respectively. The corresponding figures for women were 0.86 and 2.76 per 1000 person years. The mean age at the time of the first CE was 69 years for fatal CEs and 66 years for non-fatal events. The HRs for non-fatal and fatal first-time CEs are reported in table 2.

**Table 2:** Relationships between cardiovascular risk factors and incidence of non-fatal and fatal first-time coronary events and the significance of difference between the two outcomes

	Non-fatal coronary event			Fatal coronary event			Heterogeneity
	HR	95% CI	P value	HR	95% CI	P value	P value
**Model 1**							
Age, year	1.07	1.07 to 1.08	<0.001	1.12	1.11 to 1.13	<0.001	<0.0001
Male gender	2.54	2.33 to 2.76	<0.001	4.06	3.51 to 4.69	<0.001	<0.0001
Diabetes	2.35	2.15 to 2.57	<0.001	2.52	2.20 to 2.89	<0.001	0.57
Current smoking	1.88	1.77 to 2.01	<0.001	2.48	2.24 to 2.76	<0.001	<0.0001
Cholesterol, per mmol/L	1.32	1.29 to 1.36	<0.001	1.33	1.27 to 1.39	<0.001	0.86
Body mass index, per kg/m^2^	1.25	1.19 to 1.31	<0.001	1.43	1.32 to 1.54	<0.001	0.0031
Systolic blood pressure, per 10 mm Hg	1.14	1.12 to 1.16	<0.001	1.22	1.19 to 1.25	<0.001	0.0001
Diastolic blood pressure, per 10 mm Hg	1.20	1.16 to 1.24	<0.001	1.34	1.28 to 1.41	<0.001	0.0001
Antihypertensive treatment	1.55	1.38 to 1.75	<0.001	1.77	1.49 to 2.10	<0.001	0.23
**Model 2**							
Age, per year	1.06	1.06 to 1.07	<0.001	1.11	1.10 to 1.12	<0.001	<0.0001
Male gender	2.30	2.11 to 2.50	<0.001	3.52	3.03 to 4.08	<0.001	<0.0001
Diabetes	1.94	1.70 to 2.21	<0.001	1.90	1.56 to 2.32	<0.001	0.867
Current smoking	1.93	1.81 to 2.05	<0.001	2.68	2.41 to 2.98	<0.001	<0.0001
Cholesterol, per mmol/L	1.26	1.23 to 1.29	<0.001	1.24	1.18 to 1.29	<0.001	0.473
Body mass index, per kg/m^2^	1.14	1.08 to 1.19	<0.001	1.28	1.19 to 1.39	<0.001	0.011
Systolic blood pressure, per 10 mm Hg	1.11	1.09 to 1.13	<0.001	1.19	1.15 to 1.23	<0.001	0.0002
Diastolic blood pressure, per 10 mm Hg	1.16	1.12 to 1.20	<0.001	1.29	1.23 to 1.36	<0.001	0.0007
Antihypertensive treatment	1.32	1.17 to 1.50	<0.001	1.37	1.15 to 1.64	0.001	0.659

Model 1 includes covariates age and gender.

Model 2 includes age, gender, body mass index, diabetes, smoking habits, cholesterol, either systolic blood or diastolic blood pressure and antihypertensive treatment. Note: when systolic and diastolic blood pressure were included in the same model, systolic blood pressure showed stronger relationships to both non-fatal and fatal coronary events, compared with diastolic blood pressure.

All established risk factors were associated with both fatal and non-fatal CEs, both after adjustments for age and gender and in the multivariable model. Age (p<0.001), male gender (p<0.001), blood pressure (p<0.001), BMI (p=0.011) and smoking (p<0.001) were preferentially associated with fatal events compared with non-fatal events. Diabetes, cholesterol and antihypertensive treatment were similarly associated with fatal and non-fatal CEs (table 2).

**Figure 1:**
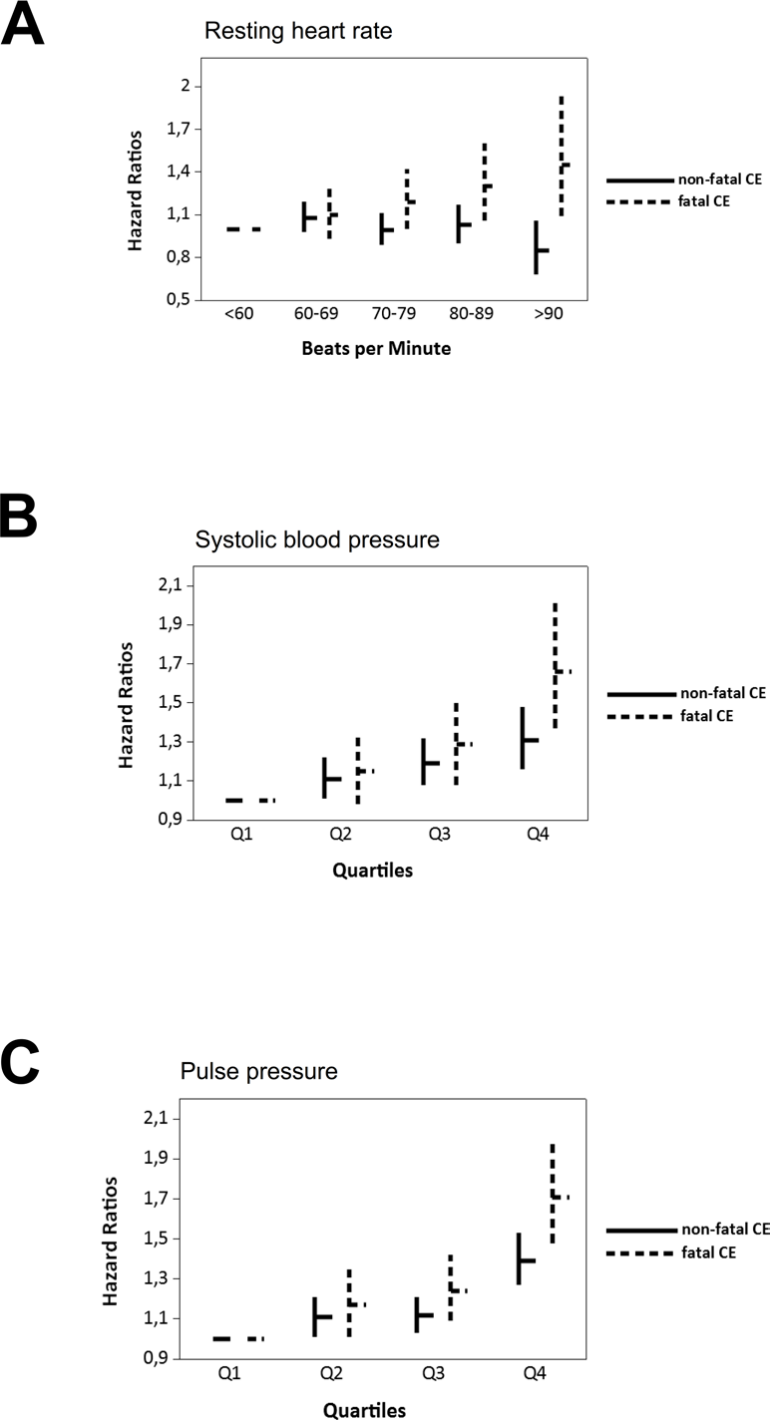
The relationship between haemodynamic measurements and incidence of non-fatal and fatal coronary events (CEs). HRs (depicted by horizontal bars) and the 95% CI (vertical bars) for non-fatal (solid lines) and fatal (dashed lines) CEs in relation to resting heart rate, systolic blood pressure and pulse pressure at baseline. Mean (range) in mm Hg for the quartiles of systolic blood pressure were 110 (75–115), 122 (117–125), 132 (128–135), 149 (137–255) for quartile 1, 2, 3 and 4, respectively. Mean (range) in mm Hg for the quartiles of pulse pressure were 31 (0–35), 40 (37–40), 47 (41–50) and 61 (51–130) for quartiles 1, 2, 3 and 4, respectively.

Orthostatic hypotension, systolic and diastolic blood pressure decline on standing, increased resting heart rate at baseline and pulse pressure were all significantly associated with both fatal and non-fatal CEs after adjustments for age and gender (table 3). The associations between orthostatic reactions and both fatal and non-fatal CEs were attenuated after adjustments for cardiovascular risk factors and no longer statistically significant. Resting heart rate (p<0.001) and pulse pressure (p=0.013) were both preferentially associated with fatal CEs (figure 1A–C).

**Table 3:** Relationships between haemodynamic parameters and incidence of non-fatal and fatal first-time coronary events and the significance of difference between the two different outcomes

	Non-fatal coronary event			Fatal coronary event			Heterogeneity
	HR	95% CI	P value	HR	95% CI	P value	P value
Orthostatic hypotension							
Model 1	1.29	1.14 to 1.45	<0.001	1.32	1.10 to 1.58	0.003	0.84
Model 2	1.09	0.96 to 1.23	0.180	1.02	0.84 to 1.23	0.860	0.56
∆SBP per −10∆mm Hg							
Model 1	1.10	1.06 to 1.14	<0.001	1.07	1.01 to 1.14	0.015	0.52
Model 2	1.03	1.00 to 1.07	0.084	0.97	0.92 to 1.03	0.364	0.089
∆DBP per −10∆mm Hg							
Model 1	1.16	1.09 to 1.23	<0.001	1.10	1.00 to 1.21	0.041	0.37
Model 2*	1.06	1.00 to 1.13	0.058	0.95	0.86 to 1.04	0.275	0.052
PP per 10 mm Hg							
Model 1	1.13	1.10 to 1.17	<0.001	1.22	1.17 to 1.27	<0.001	0.007
Model 2†	1.10	1.07 to 1.13	<0.001	1.18	1.13 to 1.23	<0.001	0.013
RHR per 10 bpm							
Model 1	1.07	1.03 to 1.10	<0.001	1.22	1.17 to 1.28	<0.001	<0.0001
Model 2	0.98	0.95 to 1.01	0.181	1.09	1.04 to 1.15	0.001	0.0004

Model 1 includes covariates age and gender.

Model 2 includes age, gender, diabetes, smoking habits, cholesterol, body mass index, antihypertensive treatment and systolic blood pressure.

*Adjusted for diastolic instead of systolic blood pressure.

†Not adjusted for systolic or diastolic blood pressure.

bpm, beats per minute; DBP, diastolic blood pressure; PP, pulse pressure; RHR, resting heart rate at baseline; SBP, systolic blood pressure.

## Discussion

In the present study, we explored which traditional risk factors and cardiovascular autonomic indices were preferentially associated with fatal CEs in the population. Age, gender, smoking, blood pressure, pulse pressure and BMI were stronger predictors of fatal CEs than non-fatal events, whereas cholesterol and diabetes showed similar associations with fatal and non-fatal CEs. Among cardiovascular autonomic indices, only higher resting heart rate was predictive of fatal CEs.

Sudden cardiac death as the first manifestation of coronary artery disease remains a major challenge in cardiovascular primary prevention.^3 4^ Only a few prospective studies have examined the effects of established cardiovascular risk factors on the fatality of CEs.^9 10 13^ Hypertension, age and male gender have been implicated in increased risk of fatal CEs,^9 10 22^ which is in line with our findings. In contrast, previous studies have reported that diabetes is a stronger risk factor for fatal than for non-fatal CEs,^10^ which was not the case in our study. Since previous studies have used 28 days and not the first day as the time frame for a fatal CE, this may indicate that diabetes is associated with poorer recovery and increased mortality after the event but not with sudden cardiac death per se. The relationships between fatality of future CEs and smoking have been inconsistent in previous studies.^9 10^

The reason for the relationship between certain established risk factors and increased fatality in CEs is not clearly understood. The mechanisms that link increased blood pressure and elevated pulse pressure to CEs and its fatality may involve endothelial damage, atherosclerosis and left ventricular hypertrophy. Stress and structural changes of the valves, atrial myocardium and left ventricular myocardium result in hypertrophy or dilatation and structural changes of the heart, which also can result in disturbances in its electrical system.^3 6^ Earlier studies have shown that left ventricular dysfunction of any cause is a strong predictor of sudden cardiac death and that tachyarrhythmia is the most common electrophysiological mechanism leading to sudden cardiac death.^3 6 23^ Hence, hypertension related to hyperadrenergic drive and neuroendocrine hyper activation can increase the risk of fatality by promoting mechanisms related to arrhythmia as well as ischaemia and atherosclerosis.

Beside established cardiovascular risk factors, manifestations indicating cardiovascular autonomic dysfunction have become increasingly recognised as important cardiovascular risk factors. Previous studies have shown increased incidence of CVD in individuals with orthostatic hypotension^14 16^ and elevated resting heart rate,^24^ measures that are believed to reflect the cardiovascular autonomic function.

Resting heart rate was significantly associated with fatal CEs in our study. This finding is largely in concordance with a study of British men that has reported an association with sudden cardiac death and a non-significant association with fatal CEs.^10 25^ Resting heart rate reflects the balance between sympathetic and parasympathetic inputs.^26^ An increased heart rate may thus indicate an autonomic state of increased sympathetic activity.^24 26^ Even though not proved by our current data, it is reasonable to hypothesise that such increased sympathetic activity may by itself increase the risk of coronary death by a number of pathophysiological mechanisms, such as increased propensity for malignant arrhythmias as well as an increased myocardial oxygen demand at the time of plaque rupture. However, elevated resting heart rate is also a non-specific marker of other health indicators that may indicate the risk of a poor outcome, such as poor physical fitness, exposure to smoking, poor lung function and overweight.^27^ Thus, a number of various factors may contribute to the observed association between elevated resting heart rate and CE fatality.

There are some important limitations of our current study. We do not have information about death within the first hour, which would have been valuable for assessing true sudden cardiac death. Moreover, the specific diagnosis of ischaemic heart disease as a cause of death always implies some degree of uncertainty, even in this context with autopsy confirmation in 60% of the population. Previous validation studies have shown acceptable validity for the diagnosis of myocardial infarction in the Swedish National Hospital Discharge Register^28^ and the Swedish Cause of Death Register.^29^ We assessed the various risk factors at baseline only, not during follow-up and/or at the time of the event. Individuals with high risk were referred for further evaluations and treatment of risk factors. A previous study reported that the interventions had no effect on mortality in the whole cohort.^17^ It is also likely any effect of intervention should be largely similar both for fatal and non-fatal events. Finally, as a methodological limitation, blood pressure on standing was measured after 1 min, not at 3 min as recommended in the current consensus of orthostatic hypotension.^18^ This reflects the fact that this consensus was not yet developed at the time of the baseline examination.

## Conclusions

In this large population-based study with a particularly long follow-up, we report that certain established cardiovascular risk factors as well as increased resting heart rate are preferentially associated with higher risk of fatal outcome during first-ever CE. These risk factors may require special attention in cardiovascular prevention programmes and in the overall efforts to further reduce coronary artery disease mortality.

## Data Availability

Data are available on reasonable request. Please contact the corresponding author.
